# Understanding the importance of motivational intensity in English as a foreign language context: A structural equation modeling analysis

**DOI:** 10.3389/fpsyg.2022.1020558

**Published:** 2022-11-15

**Authors:** Cong Wang, Sida Zhu, Haijing Zhang

**Affiliations:** Department of Chinese Language Studies, The Education University of Hong Kong, Tai Po, Hong Kong SAR, China

**Keywords:** motivational intensity, learning motivation, learning English as a foreign language, structural equation modeling, Hong Kong university students

## Abstract

**Introduction:**

Motivational intensity is the effort learners make in language learning. It is an essential component and a direct measurement of L2 motivation. Few studies have distinguished motivational intensity from motivation and explored its role in learning English as a foreign language (EFL).

**Methods:**

This study examined 208 university students from Hong Kong to investigate the factors that affect motivational intensity and explored the relationship between motivational intensity and learning motivation using structural equation modeling (SEM).

**Results and discussion:**

The students’ motivational intensity was affected by personal factors (daily English-learning time and stage of English learning), family factors (monthly household income and parental attitudes), and school factors (English learning engagement and satisfaction). The differences in personal factors, school factors and monthly household income of family factors among different motivational intensity groups were significant whereas the difference in parental attitudes (family factors) between the high- and the low-motivational intensity groups was insignificant. As for the relationship between motivational intensity and motivation, motivational intensity indirectly affected students’ intrinsic interest through their attitudes toward native English speakers (β = 0.16, *p* = 0.041 < 0.05). The significant path coefficient from the learning situation to attitudes toward native speakers was negative (*p* < 0.05), indicating that attitudes toward native speakers decline even when the learning situation improves. This study enriched the theoretical study of motivation theory and provided teaching suggestions for improving EFL learning.

## Introduction

Motivation plays an essential role in learning English as a foreign language (EFL). Improving motivation level can reduce the EFL learners’ anxiety and promote their English learning effectiveness (Kazemi et al., 2020; Alamer and Al Khateeb, 2021; Yang and Wang, 2021; Dewaele et al., 2022; Mahmoodi and Yousefi, 2022; Norouzifard et al., 2022). Motivation is a dynamic process of changes that contains a learner’s learning effort, cognition, and affect (Lalonde and Gardner, 1985). Motivational intensity is the degree of effort learners make to achieve their learning goals, which is affected by learners’ cognitive level and related to learners’ emotions and family backgrounds (Filetti et al., 2019). Questionnaires are used as the primary research instrument to measure learners’ motivational intensity (Ding, 2014). Gable and Harmon-Jones (2010) divided learners’ motivational intensity into low-intensity, medium-intensity, and high-intensity to analyze the variations of motivation in English learning among individuals. To improve EFL learners’ learning effectiveness and efficiency, understanding the effort learners have made in English learning is of great importance, i.e., it is necessary to investigate variations in the motivational intensity of EFL learners and analyze the factors that affect motivational intensity.

This study adopted a quantitative research method to investigate the factors that affect motivational intensity with the evidence from EFL university students from Hong Kong. The correlation analysis and one-way analysis of variance (ANOVA) in SPSS.26 were adopted to investigate the influencing factors of EFL learners’ motivational intensity and to explore the differences of these factors in different motivational intensity groups. Furthermore, the maximum likelihood was checked to ensure that the various indicators of the model were within a reasonable range before the structural equation modeling (SEM) model was constructed by AMOS.26. Finally, the model was analyzed to discuss the interaction among different variables and explore the relationship between motivational intensity and different motivation variables. This study distinguished motivational intensity from motivation and explored its importance in EFL learning, which enriched the theoretical study of motivation theory and provided new evidence for studies on EFL. In addition, this study analyzed individual differences in the factors affecting EFL learners’ learning efforts, which is conducive to clarifying the role of multiple factors in EFL learning and providing teaching suggestions for improving EFL learning effectiveness.

## Literature review

### Motivational intensity and its affecting factors

Learning motivation refers to a combination of a learner’s investment, desire to learn, and language attitudes in learning (Gardner et al., 2004). In second language (L2) learning, EFL learning in particular, motivation dramatically affects the learning effectiveness and learners’ willingness to communicate in the target language. It determines the learners engagement in L2 learning and confidence in overcoming difficulties (Oxford et al., 2004). Motivation plays an essential role in EFL learners’ participation in English courses and maintaining a high level of personal effort (Dörnyei, 2001; Zimmerman, 2011). Motivation affects the learning achievement of EFL learners as well as individual learning behavior and strategies (Jang and Lee, 2019). Highly motivated EFL learners are generally outstanding in academics and do not feel anxious and uneasy in English learning (Carreira, 2011), while low motivation is considered as the main factor that reduces EFL learners’ learning effectiveness (Lan, 2015). A high motivation for L2 learning, which indicates a high degree of effort spent on learning the language (Lalonde and Gardner, 1985), is accompanied by remarkable persistence in L2 learning (Mori and Gobel, 2021). English learners’ level of motivation is affected by their intrinsic interest, which enhances their pragmatic awareness and promotes their cultivating motivation (Li et al., 2015). SEM has been used to explore the internal structure of motivation and to explain the relationships among different motivation variables by analyzing differences between motivation and factors such as L2 proficiency (Hu and Ma, 2019; Fathi and Mohammaddokht, 2021).

L2 motivation includes three components: motivational intensity (effort), desire to learn a language (cognition), and attitudes toward language learning (affect) (Gardner, 1985). Motivational intensity refers to learners’ effort in L2 learning, which impacts the goals, self-efficacy, and effectiveness of and attitudes toward language learning (Tremblay and Gardner, 1995). Motivational intensity is marked by differences among individuals and significantly correlates with gender, socio-economic status, and educational background. It negatively correlates with classroom anxiety in the EFL context (Lalonde and Gardner, 1985; Gardner et al., 1997). For example, Hong Kong females’ internal motivation for language learning is significantly higher than males’, and males’ motivational intensity is lower than females’ (Anderman and Midgley, 1997; Lau, 2009). Learners’ motivational intensity is affected by the family environment, including household income and parental support (Wong, 2007, 2010), teachers’ support, and the participating time in learning activities (Wong, 2007; Lee et al., 2009). The length of learning time and differences in language competence may also lead to differences in motivational intensity (Wong, 2007). University students’ English learning achievement is significantly correlated with their motivational intensity, attitudes, desires to learn English, and perception of native English speakers and the quality of English courses (Cocca and Cocca, 2019). Internal factors such as personal willingness and language competence affect the motivational intensity in language learning, and so do external factors, including parents’ and teachers’ impact, and material/spiritual rewards (Chen et al., 2018; Martin, 2022). Affected by factors such as study pressure, the motivational intensity of some students who enter university with higher motivation to learn English declines over time (Thang et al., 2011).

Motivational intensity as the effort at the behavioral level plays an important role in and has a significant correlation with the level of learning motivation (Dörnyei, 1994; Tremblay and Gardner, 1995; Csizér and Kormos, 2008). Other components of motivation significantly affect L2 learners’ motivational intensity (Gardner et al., 2004). Attitudes toward an L2 significantly predicts the motivational intensity (Hermessi, 2022), and so do learners’ effort level, ideal L2 self/own, and ought to L2 self/own (Feng and Papi, 2020). Changes in students’ attitudes toward English learning may determine whether they will increase their English contact in activities such as studying or job hunting, affecting their effort in L2 learning (Wong, 2010). In general, motivational intensity, a critical variable in L2 learning, is highly correlated with different motivation variables.

### The role of motivational intensity in second language learning

Motivational intensity is regarded as the goal-oriented effort of learners to learn a foreign language (Ellis, 2004). It plays a vital role in motivation because other components of motivation, namely attitudes, and self-efficacy, can be transformed into learning achievement through learners’ effort at the behavioral level (Tremblay and Gardner, 1995). The evidence of motivation exists in the learner’s learning behavior. Therefore, the “desire” measure is important, which directly probes into the learner’s willingness to take action. Motivational intensity, which explicitly concentrates on behaviors triggered by motivation, is a more direct measurement (Dörnyei, 1994, 2001). The motivational intensity scale may provide a suitable measure of the construct’s role in L2 learning (Gardner and MacIntyre, 1993). Motivational intensity plays a vital role in the success of L2 learning (Wong, 2007; Lee et al., 2009; Kim and Shin, 2021; Somers and Llinares, 2021). A significant correlation was found between the desire and the motivational intensity of EFL learners who plan to study abroad. In contrast, the desire and the motivational intensity before departure were significantly related to their language listening and reading ability (Mori and Gobel, 2021). Therefore, motivational intensity is important in measuring EFL learners’ motivation levels (Papi and Abdollahzadeh, 2012). Motivational intensity can affect the learning performance and the achievement of L2 learners, and this impact is associated with the overall motivation (Bernaus and Gardner, 2008; Mori and Gobel, 2021).

To accurately test L2 learners’ motivation, especially their efforts and investment, *the Attitude/Motivation Test Battery (AMTB)* was developed (Lalonde and Gardner, 1985; Gardner and MacIntyre, 1993; Gardner et al., 2004). The scale included five categories: integrativeness, attitudes toward the learning situation, motivation, language anxiety, and other attributes (Gardner et al., 1997). Integrativeness refers to L2 learners’ willingness to communicate with others, covering attitudes toward the target language group, interest in the target language, and comprehensive orientation for language learning. Attitudes toward the learning situation are L2 learners’ attitudes and reactions toward L2 teaching, i.e., their attitudes toward teachers and language courses. Motivation consists of attitudes to language learning, desire to learn an L2, and motivational intensity. Language anxiety reflects an individual’s awareness of L2 learning and environment. Other attributes indicate other variables in L2 learning, usually measured by the *Instrumental Orientation scale* (Gardner, 1985). These variables in AMTB are correlated with L2 learners’ performance (Zimmerman, 2011; Mori and Gobel, 2021). Learners’ effort, attitudes, and willingness which closely correlate with L2 performance are more significant than other variables (Gardner, 1985). Therefore, this study used AMTB as a tool to analyze the motivation of L2 learners.

English is the L2 for most Hong Kong university students whose English learning achievement varies because of the differences in motivation (Li and Leung, 2020). Improving English learning achievement of Hong Kong EFL students has become a core issue (Yu et al., 2016). Some studies have analyzed the motivation levels of EFL students in Hong Kong and discussed the significant differences in various factors, whereas few studies have taken family, school, and personal perspectives into consideration when analyzing factors affecting motivation (Bai and Guo, 2018; Shen et al., 2020). Previous research did not clarify the features of changes in motivation (increase or decrease) in the EFL learning process, neither did they discuss the factors that affect the changes at different motivation levels (Ehrman, 1996; Schmidt et al., 1996). In terms of research methods, most studies adopted the analysis of variance method in SPSS, which can neither accurately explain the overall structure of the internal factors of motivation, nor describe the correlation between the factors (Dörnyei, 2001; Zimmerman, 2011). However, correlation, factor analysis, and analysis of variance were taken as the primary research methods for analyzing the relationship between the motivational intensity and the motivation in the context of EFL (e.g., Ehrman, 1996; Schmidt et al., 1996; Ding, 2014; Filetti et al., 2019; Hermessi, 2022). Besides, previous studies mainly focused on model validation between motivation and the expected efforts of learners other than learners’ actual effort (Hu and Ma, 2019) and did not strictly distinguish motivational intensity from learning motivation (Dörnyei, 1994, 2001; Zimmerman, 2011; Yu et al., 2016; Mori and Gobel, 2021). To fill the research gap, this study differentiated motivational intensity from learning motivation and took Hong Kong university students as an example to explore the role of motivational intensity in EFL learning.

### The present study

Referring to the AMTB and the motivational intensity scale (Gardner and MacIntyre, 1993), this study surveyed EFL students from eight public universities in Hong Kong. Aiming to explore the changing regular of internal and external factors for motivational intensity, tests of between-subject effects were conducted to investigate the influencing factors with different degrees of motivational intensity. Then, the relationship between motivational intensity and learning motivation was analyzed by SEM. After multiple revisions, a SEM model was constructed, which revealed the influence of motivational intensity on overall learning motivation. Finally, we further analyzed the differences between the two and explored the direct and indirect effects of motivational intensity and different motivation variables.

This research aimed to address the following research questions (RQs):

RQ1. What factors affect the motivational intensity of English learning among Hong Kong university students?
RQ2. What are the differences between these factors in different motivational intensity groups (low-intensity, medium-intensity, and high-intensity)?
RQ3. What is the relationship between motivational intensity and different motivation variables?

## Methodology

### Participants

Initially, 249 full time undergraduates (*N* = 249, Female = 150, Male = 99) aged between 18 and 22 (*M* = 20.57, *SD* = 0.95) from eight public universities in Hong Kong were recruited. All these students are non-English majors from different programs. Among them, 71.9% have studied English for more than 11 years, 18% for 5–11 years, and the rest for less than 5 years. Over 55% spend more than half an hour on English learning every day, and more than 78% reported high degrees of family support for English learning. To ensure the unity of the participants’ native language background, the non-Cantonese speakers (*N* = 41) were excluded. Finally, 208 Cantonese EFL learners (*N* = 208, Female = 120, Male = 88, Mean of age = 20.37, SD of age = 0.78) were considered as valid participants for this study.

### Instrument

The questionnaire consisted of three parts (Q1, Q2, and Q3) and 77 compulsory items. Q1 is developed to collect the background information, including 24 items with 14 items capturing personal factors (e.g., gender, years of learning English, learning experience in English native speaking countries), eight items focusing on family factors (e.g., parents’ education backgrounds and English proficiency) and two related to school factors (e.g., English learning satisfaction). Q2 was entitled *Learning Motivation*, adopted from *the AMTB* (Gardner and MacIntyre, 1993). It consisted of 42 items, including items 1–9 addressing EFL learners’ attitudes toward English native speakers (e.g., After learning English, I have become more and more inclined to communicate with foreigners in English); items 11–19 focusing on EFL learners’ intrinsic interest (e.g., I can enjoy the process of learning English); items 20–23 exploring the perception of English culture (e.g., I want to understand foreign culture and art, so learning English is very important); items 24–27 seeking for EFL learners’ ultimate goals (e.g., Learning English is very important because I want to conduct research in English); items 28–32 concerning EFL learners’ learning environment (e.g., When I speak in an English class, unfamiliar content makes me lack confidence); and items 33–42 involving parental influence (e.g., My parents helped me to learn English as much as possible). Q3 was *The Motivational Intensity* adopted from *The Motivational Intensity Scale* (Gardner and MacIntyre, 1993). It contained 11 items to investigate the motivational intensity for English learning (e.g., I take the initiative to read English newspapers outside of class and pay attention to the use of English in them). To reduce the effort to understand each item, all the items were presented in Chinese and English.

Items in Q1 are multiple-choice, requiring participants to choose an appropriate item based on their situations. Q2 and Q3 are 5-point Likert scales (*1* = *never, 2* = *rarely, 3* = *sometimes, 4* = *often, 5* = *always*) that required participants to select the option corresponding to their situations. The reliability of Q2 and Q3 was analyzed by SPSS.26, and Cronbach’s α value was tested. The results showed that the reliability of Q2 and Q3 were 0.888 and 0.817, respectively, both greater than 0.8, indicating high reliabilities (Kaiser, 1974).

### Data analysis

The questionnaire was distributed to the participants *via* Google Forms, and a maximum of 35 minutes was given. Submissions exceeding 35 min would not be accepted. All subjects completed the questionnaire within the prescribed time, and 208 valid questionnaire responses were collected, which were processed and analyzed by SPSS.26 and AMOS.26. *Bivariate correlations* and *Pearson coefficient* were selected in SPSS.26 to test factors affecting motivational intensity (RQ1).

The *Descriptive statistics* and *Reports* in SPSS.26 were used to compute the number of different motivational intensity groups: the descriptive groups of low intensity, medium intensity, and high intensity by referring to the classification standards of previous scholars (Oxford and Burry-Stock, 1995). Combined with *one-way ANOVA*, the influence of different factors on Hong Kong university students’ motivational intensity was investigated (RQ2).

**FIGURE 1:**
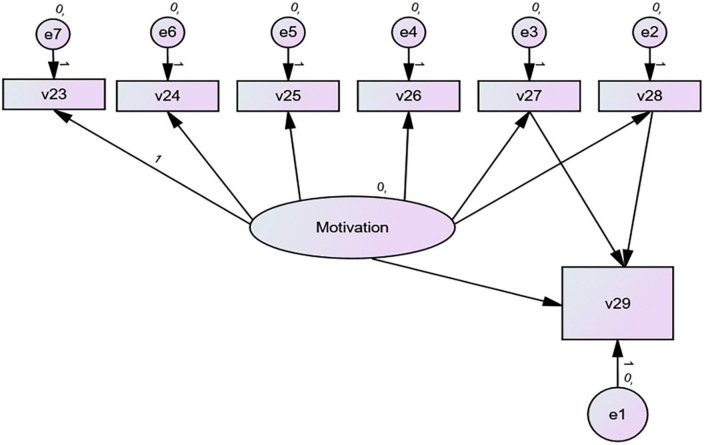
The default model. V23, attitudes toward English native speakers; V24, intrinsic interest; V25, English culture; V26, ultimate goal; V27, learning situation; V28, parental encouragement; V29, motivational intensity.

Then, AMOS.26 was used to establish the SEM model to investigate the influence of motivational intensity on other motivation variables (RQ3). According to Gardner (1985), a conceptual model was established (see Figure 1). This conceptual model attempted to uncover the relationship between motivation and motivational intensity, i.e., how various motivation variables related to motivational intensity. For example, learning situation, parental engagement, and the overall motivation could predict motivational intensity positively. A well-fit model needs to meet the requirement that multiple indicators (CFI, GFI, IFI, NFI, etc.) should be greater than 0.9, and the path coefficient must be significant, i.e., *p* > 0.05. Therefore, the fitness of the data to the model was checked. If a low index value occurred, the model’s path would be adjusted and modified according to Byrne (2010). When a final model with all indicators that met the relevant requirements was obtained, this study analyzed the relationship between motivational intensity and different motivation variables.

## Results

### Factors affecting different motivational intensity groups

To analyze the factors affecting the motivational intensity of Hong Kong university students (RQ1), this study classified motivational intensity into three categories referring to previous classification criteria (Oxford and Burry-Stock, 1995): the high-intensity group [3.5 ≤ averages (A) ≤ 5.0], the medium-intensity group (2.5 ≤ A < 3.5) and the low-intensity group (0 ≤ A < 2.5). The result showed that approximately 26, 130, and 52 Hong Kong university students belonged to the low-, medium- and high-intensity groups, accounting for 12.5, 62.5, and 25%, respectively. Then, factors that affect the motivational intensity for English learning were investigated. According to the results of the *Pearson correlation* on *bivariate correlations*, there were six significant factors (*p* < 0.05), including two personal factors (daily English-learning time and stage of English learning), two family factors (monthly household income and parental attitudes), and two school factors (English learning engagement and satisfaction) (see Tables 1, 2). All the factors were positively correlated with motivational intensity except the stage of English learning, which was negatively correlated with motivational intensity (*Pearson correlation* = −0.174).

**TABLE 1:** Descriptive.

Factor	Group	Mean	SD	SE
Daily English-learning time	Low	1.5484	0.85005	0.15267
	Medium	1.8319	1.00803	0.09483
	High	2.375	1.16155	0.14519
Stage of English learning	Low	2.0645	0.44238	0.07945
	Medium	2.0796	0.53688	0.05051
	High	1.8281	0.57885	0.07236
Monthly household income	Low	2.2258	0.88354	0.15869
	Medium	2.7788	0.97956	0.09215
	High	2.7656	1.1918	0.14897
Parental attitudes	Low	4.1935	0.87252	0.15671
	Medium	4.0619	0.82682	0.07778
	High	4.5469	0.68845	0.08606
English learning engagement	Low	2.2581	0.85509	0.15358
	Medium	2.5841	0.83158	0.07823
	High	3.0625	0.85217	0.10652
English learning satisfaction	Low	2.129	0.99136	0.17805
	Medium	2.708	0.85232	0.08018
	High	3.25	0.85449	0.10681

**TABLE 2:** *Post hoc*.

	Group of the motivational intensity		Mean	Sig.	95% confidence interval	
			difference			
					Lower bound	Upper bound
Daily English-learning time	Low	Medium	–0.28347	0.179	–0.698	0.1311
		High	−0.82661*	0	–1.274	–0.3792
	Medium	Low	0.28347	0.179	–0.1311	0.698
		High	−0.54314*	0.001	–0.863	–0.2233
	High	Low	0.76343*	0.003	0.3792	1.274
		Medium	0.40266*	0.02	0.2233	0.863
Stage of English learning	Low	Medium	–0.01513	0.89	–0.2301	0.1998
		High	0.23639*	0.046	0.0044	0.4684
	Medium	Low	0.01513	0.89	–0.1998	0.2301
		High	0.25152*	0.003	0.0857	0.4174
	High	Low	–0.2656	0.041	–0.4684	–0.0044
		Medium	–0.25069	0.005	–0.4174	–0.0857
Monthly household income	Low	Medium	−0.59034*	0.009	–0.9674	–0.1385
		High	−0.086*	0.007	–0.9871	–0.0925
	Medium	Low	0.55295*	0.009	0.1385	0.9674
		High	0.1314	0.936	–0.3067	0.3329
	High	Low	0.53982*	0.018	0.0925	0.9871
		Medium	–0.01314	0.936	–0.332	0.3067
Parental attitudes	Low	Medium	−0.55295*	0.009	–0.1858	0.449
		High	−0.53982*	0.018	–0.6959	–0.0107
	Medium	Low	0.55295*	0.009	–0.449	0.1858
		High	0.1314	0.936	–0.7299	–0.24
	High	Low	0.37373	0.052	0.0107	0.6959
		Medium	0.46526*	0	0.24	0.7299
English learning engagement	Low	Medium	–0.32601	0.57	–0.6624	0.0103
		High	−0.80444*	0	–1.1674	–0.4414
	Medium	Low	0.32601	0.57	–0.0103	0.6624
		High	−0.47843*	0	–0.738	–0.2189
	High	Low	1.05443*	0	0.4414	1.1674
		Medium	0.57291*	0	0.2189	0.738
English learning satisfaction	Low	Medium	−0.57893*	0.001	–0.9286	–0.2293
		High	1.12097*	0	–1.4983	–0.7436
	Medium	Low	0.57893*	0.001	0.2293	0.9286
		High	0.54204*	0	–0.8118	–0.2722
	High	Low	1.22496*	0	0.7436	1.4983
		Medium	0.58096*	0	0.2722	0.8118

*The mean difference is significant when *p* < 0.05.

To explore the differences in affecting factors among different motivational intensity groups (RQ2), *the post hoc test* in the *one-way ANOVA* was performed. According to the results of *multiple comparisons:* the mean difference of daily English-learning time between the high and the medium-intensity groups and the high and the low-intensity groups increased with the improvement of the motivational intensity. In terms of the stage of English learning, the mean difference between the high and the medium-intensity groups was −0.25069 (*p* < 0.05), while the mean difference between the high and the medium-intensity groups was −0.26560 (*p* < 0.05), demonstrating a negative correlation between the stage of English learning and motivational intensity. The mean difference between the high and the medium-intensity groups in parental attitudes was 0.46526 (*p* < 0.05), whereas no significant difference between the high and the low-intensity groups was found (*p* = 0.052 > 0.05). The mean difference increased with a continuous rise of the motivational intensity regarding monthly household income, from −0.59034 (low- and medium-intensity groups) to −0.08600 (low- and high-intensity groups). Although there was a negative correlation between household income and motivational intensity, the mean difference increased with motivational intensity. As for the two school factors (English learning engagement and satisfaction), the mean differences among different motivational intensity groups (high- and medium-intensity groups; high- and low-intensity groups) were positive, suggesting that both school factors positively correlated with EFL learners’ motivational intensity.

### Influence of motivational intensity on learning motivation

**FIGURE 2:**
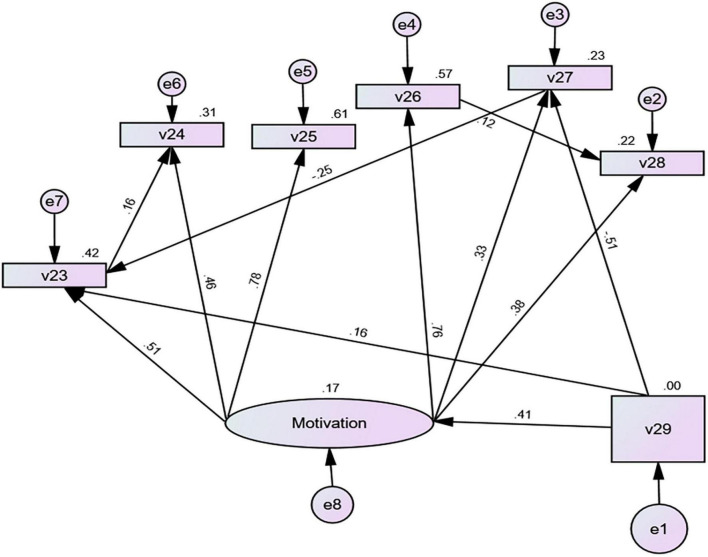
The SEM model of relationships between the motivational intensity and learning motivation. V23, attitudes toward native speakers of English; V24, intrinsic interest; V25, English culture; V26, ultimate goal; V27, learning situation; V28, parental encouragement; V29, motivational intensity.

Some research demonstrates that motivational intensity affects students’ motivation (such as reducing or increasing motivation), varying in learners internal and external factors (Wong, 2007). This study explored the relationship between motivational intensity and different motivation variables by constructing a SEM model (RQ3). As the fit indices of the data and model were not acceptable, the default model (see Figure 1) needed further modification. After revising the default model, we found adequate fit for this model’s re-specification including all variables (CMIN/DF = 1.337 < 2, CFI = 0.991 > 0.9, GFI = 0.984 > 0.9, IFI = 0.992 > 0.9, NFI = 0.968 > 0.9, TLI = 0.980 > 0.9, RMSEA = 0.040 < 0.05) (see Figure 2). Table 3 shows the result obtained from AMOS 26. Model fit indices meet acceptable levels (CMIN/DF < 2, CFI, GFI, IFI, NFI, TLI ≥ 0.9, RMSEA ≤ 0.05), indicating that this model can be accepted (Browne and Cudeck, 1993; Cheung and Rensvold, 2002).

**TABLE 3:** Model fit indices.

	CMIN/DF	CFI	GFI	IFI	NFI	TLI	RMSEA
Model respecification	1.337	0.991	0.984	0.992	0.968	0.980	0.040

According to Figure 2, the standardized path coefficient from motivational intensity to learning motivation was 0.41 and *p* < 0.001, indicating a positive correlation between motivational intensity and learning motivation. The standardized path coefficient from motivational intensity to learning situation was −0.51 (*p* < 0.001), implying a negative correlation-the greater the motivational intensity, the lower the learning situation. The path coefficient from motivational intensity to attitudes toward English native speakers was 0.16 (*p* = 0.031 < 0.05), showing that increased motivational intensity was accompanied by grown attitudes toward English native speakers. Motivational intensity indirectly affected students’ intrinsic interest through their attitudes toward English native speakers (β = 0.16, *p* = 0.041 < 0.05). Path coefficients between different motivation variables were also significant. A significant path coefficient from learning situation to attitudes toward English native speakers was −0.252, indicating that attitudes toward English native speakers decreased even when the learning situation was improved.

## Discussion

### Influencing factors and the variations of them in different motivational intensity groups

#### Personal factors in different motivational intensity groups

The Hong Kong Diploma of Secondary Education is a compulsory exam for Hong Kong students who wish to enter universities in Hong Kong. Students with higher scores in that exam have more choices in various programs when entering the university. The *post hoc analysis* was conducted, and no correlation between motivational intensity and diploma score was found. The stage of English learning showed a significant difference in motivational intensity. Moreover, the mean differences between the high- and the medium-intensity groups and between the high- and the low-intensity groups were both negative. Motivational intensity showed a significant downward trend at advancing stages of English learning. Namely, Hong Kong university students at lower English learning stages have higher motivational intensity. Differences among the motivational intensity groups also significantly affected daily English-learning time in two aspects. The mean difference was 0.76343 between the high- and the low-intensity groups, while the mean difference was −0.40266 between the high- and the medium-intensity groups, indicating that daily English-learning time can affect Hong Kong university students’ motivational intensity; that is, their motivational intensity increases with daily English-learning time.

The finding that learners’ person factors generally impact their motivational intensity is consistent with previous research (Yu et al., 2018; Mori and Gobel, 2021; Nguyen and Habók, 2021; Dewaele et al., 2022; Hermessi, 2022). The correlation between daily English-learning time and motivational intensity is positive while there is a negative correlation between the stage of English learning and motivational intensity. In terms of daily English-learning time and the stage of English learning, the mean differences among different motivational intensity groups (high- and medium-intensity groups; high- and low-intensity groups) showed a regular change, which is consistent with the change of motivational intensity found in previous research (Noels et al., 2019; Dewaele et al., 2022; Xie et al., 2022). Besides, a negative correlation between the stage of English learning and motivational intensity implied that students learning English earlier would have higher motivational intensity than those who learn English relatively late. This phenomenon may be related to personal interest or family education background. Such a result differs from previous studies on university students in Hong Kong (Li and Leung, 2020). As the importance of learning English is highly recognized in Hong Kong, and it is an L2 to which university students in Hong Kong have been exposed for a long time (Tung, 1998), some researchers believe that learners’ motivational intensity was positively correlated with the stage of English learning (Tsang, 2019; Li and Leung, 2020).

In addition, students who spend more time on English learning have higher motivational intensity, because of either cognition or external pressures such as continuing education and job hunting. It is consistent with previous studies that most university students in Hong Kong are goal-oriented when learning English with the precise aim of job hunting/enrollment for higher education, so they made massive efforts in English learning (Li and Leung, 2020; Wang et al., 2021). Changes in personal factors at different stages are the main factors affecting motivational intensity, indicating that Hong Kong university students should pay attention to their daily English-learning time, including planning their learning time reasonably and enhancing their motivational intensity for English learning.

#### Family factors in different motivational intensity groups

In terms of monthly household income, the value of the mean difference between the low-intensity group and the other two groups was negative and significant (*p* < 0.05). It means that despite significant gaps in monthly household income among different Hong Kong families, students’ motivational intensity increases with household income. As for parental attitudes, there was a positive correlation between the high and the medium-intensity groups, demonstrating that monthly household income and parental attitudes directly impact the motivational intensity for English learning. Parents’ incentives can influence language learning, such as improving their children’s confidence through self-discipline (Pfenninger and Singleton, 2019; Martin, 2022). Furthermore, parental attitudes toward language learning affect learners’ motivation, attitudes, and achievement (Chen et al., 2018; Getie, 2020). It verifies Gardner’s (1968) and Wentzel’s (1998) views. Even if parents lack appropriate language skills, their attitudes toward language and culture will also affect their children’s language attitudes. In other words, family background plays a vital role in developing students’ learning motivation. In addition, monthly household income, which refers to the total income of each family (Popa and Salant̨ă, 2017; Martin, 2022), should not be ignored. According to the results, even families with low income have a high degree of support for students’ English learning.

The reasons for differences in family factors are closely related to parents’ knowledge and social-economic status. Parents considerably impact their children’s learning motivation (Garn et al., 2010; Yang and Wang, 2021; Xia et al., 2022). Students become more enthusiastic about learning and enjoy learning English more when their parents support them (Ibabe, 2016; Zhang and Wang, 2020). This result corroborates previous studies on some level. Namely, parental attitudes significantly impact Hong Kong university students’ English learning (Li and Leung, 2020). In Hong Kong, parents provide their children with opportunities to learn English at a very early stage and support them to maintain English learning in university (Lai, 2013; Shen et al., 2020), which is a common phenomenon among Hong Kong households with different incomes (Wang et al., 2021).

More than 50% of parents have only received primary or secondary education, and their monthly household income is below 50,000 Hong Kong dollars. Combined with the special status of English in Hong Kong, this implies that most Hong Kong families have disadvantages in income and educational background. However, they support their children’s English learning. Teachers need to consider different factors for different students. For students with sufficient support from parents who act as supervisors to help students establish a better learning environment, English learning is a collaboration between schooling and family guidance. For students lacking family support, teachers need to exploit school resources, to appropriately increase the learning challenge and to provide students with more opportunities to experience English culture so that the students can better understand the importance of learning English. As for families with neutral attitudes toward English learning, teachers can encourage parents and students to participate in extra-curricular learning activities together so as to increase parents’ support.

#### School factors in different motivational intensity groups

For the two school factors, English learning engagement and satisfaction, the mean value differed among different motivational intensity groups. English learning engagement and satisfaction are the main factors affecting L2 learning (Alamer and Al Khateeb, 2021; Papi and Khajavy, 2021; Mahmoodi and Yousefi, 2022; Teimouri et al., 2022). With increasing times of participation in English activities and rising English learning satisfaction, Hong Kong university students’ motivational intensity has increased. In terms of English learning satisfaction, there were significant differences among the three motivational intensity groups with mean differences of 1.12097 (between the low- and the high-intensity groups and 0.54204 (between the medium and the high-intensity groups), respectively, indicating motivational intensity increased with the degree of English learning satisfaction. It is also consistent with the result of previous studies (Tung, 1998; Lee et al., 2009; Yu et al., 2016; Tsang, 2019) that satisfaction plays an important role in English learning for Hong Kong students. When highly satisfied with the learning environment, teaching methods, learning activities, etc., Hong Kong university students are self-driven to invest more effort in English learning, which promotes motivational intensity (Lee et al., 2009; Lai, 2013).

The significance of the school factors in the results indicated that teachers are crucial in the EFL, which supports the previous studies that teachers are the main factor affecting students’ success in language learning (Gan et al., 2019; Honarzad and Rassaei, 2019; Tsang, 2019; Jiang et al., 2021). To give full play to teachers’ vital role, this study suggests that they should organize various student-oriented activities by utilizing motivational strategies that increase the attractiveness of learning tasks, promote students’ active involvement, and enhance learners’ motivation. Teachers should also take advantage of classroom teaching, assessment, and other methods to cultivate students’ learning motivation to ensure students’ enthusiastic participation. In a word, it is necessary for teachers to pay more attention to the differences in motivation among EFL students, to design or arrange learning activities that match their motivational profiles to increase EFL students’ interest, and to adequately guide students to participate in English learning activities actively.

Most Hong Kong university students showed high engagement in English learning, similar to previous studies (Li and Leung, 2020). Engagement refers to the participation in English learning activities (Noels et al., 2019). Hong Kong university students actively participate in various English learning activities held by schools, suggesting a high motivational intensity in English learning. Such a situation indicates that university students in Hong Kong are highly interested in English learning activities and willing to make more efforts in English learning to ensure that their English level can meet the needs of future education and employment.

This study found that the majority of Hong Kong university students’ English learning motivation belongs to the medium or the high-intensity groups. Students pay more attention to English learning and have high motivational intensity under entrance examinations, employment, and public examinations. With increased daily English-learning time, the motivational intensity for individual language learning increases. Since internal motivation comes from a learner’s interest in a task, it is necessary to study the influence of personal internal factors on the motivational intensity from the perspective of dynamic development (Deci and Ryan, 1985). In addition, consistent with previous studies, we found that external factors such as monthly household income level and English learning satisfaction can also affect the strength of motivation (Lee et al., 2009; Yu et al., 2018; Vergara-Morales and Del Valle, 2021). Students’ extrinsic motivation will change with varying stimuli, affecting individuals’ internal motivation (Van den Broeck et al., 2021). To enhance the motivational intensity for English learning among Hong Kong university students, internal factors such as individual language level and the stage of English learning are essential, and so is the impact of change in external factors on learners.

### The relationship between motivational intensity and learning motivation

According to the SEM model, which demonstrated a significant influence of motivational intensity on the six motivation types (*p* < 0.001), there are significant differences in the motivational intensity among Hong Kong university students. It is consistent with previous research (Tremblay and Gardner, 1995; Vandergrift, 2005; Li and Leung, 2020; Mori and Gobel, 2021). Further analysis shows that the standardized path coefficient of motivational intensity for learning situation is −0.51 (*p* < 0.001), indicating a negative correlation. The higher the motivational intensity, the lower the learning situation, where “learning situation” involves the degree of influence of external conditions on learners in English learning, such as teachers, classmates, classroom environment, etc. (Gardner and MacIntyre, 1993). This result contradicts previous studies (Deci and Ryan, 1985; Lee et al., 2009; Lai, 2013). For Hong Kong university students with high motivational intensity, their attention to learning situations may decrease significantly. That is, Hong Kong university students with high investment and effort in English learning will pay more attention to the results of language learning. Changes in the external learning situations, such as adjustment of curriculum settings and increase or decrease in learning activities, will not affect Hong Kong university students’ English learning significantly. Therefore, this phenomenon further indicates that with the continuous improvement of English learning motivational intensity, the intervention effect of the external environment on university students will reduce.

In cognition, regardless of external conditions (positive or negative), the high-intensity group gradually narrows its scope of cognition, while the low-intensity group gradually expands its scope (Harmon-Jones et al., 2013). Papi and Abdollahzadeh (2012) pointed out that in the process of L2 learning, the self-awareness of low intensity is higher than that of other groups, possibly causing motivational intensity to be triggered by individual goals (Gable and Harmon-Jones, 2010). Due to differences in individual learning goals, the 208 Hong Kong university students participating in our study had differences in motivational intensity when facing external learning situations. In addition, there were significant standardized path coefficients for motivational intensity, attitudes toward native speakers of English, and intrinsic interest, all positively correlated, consistent with the results of Csizér and Kormos (2008). Thus, motivational intensity can indirectly affect intrinsic interest through attitudes toward native speakers of English, reflecting those external conditions, to a certain extent, affect learners’ imagination of L2 characteristics (Dörnyei, 2009). When Hong Kong university students are supplied with a better external environment, their cognitive range and ability increase, as a result of which they will formulate clearer study plans that can arouse stronger intrinsic interest in English learning.

## Conclusion

This study adopted a quantitative research method and investigated 208 non-English major university students from eight Hong Kong Universities to explore the importance of motivational intensity in EFL learning through One Way ANOVA and SEM. The factors that influence motivational intensity include daily English-learning time and the stage of English learning (personal factors), monthly household income and parental attitudes (family factors) and English learning engagement and satisfaction (school factors). Personal factors, school factors and monthly household income (one of family factors) differed significantly among students with different motivational intensity levels (*p* < 0.05), while there was no significant difference in parental attitudes (one of family factors) between the high- and the medium- intensity groups (*p* = 0.46526 > 0.05) and between the high- and the low- intensity groups (*p* = 0.052 > 0.05). Motivational intensity indirectly affects students’ intrinsic interest through their attitudes toward native English speakers. The significant path coefficient from the learning situation to attitudes toward native speakers is negative, indicating that attitudes toward native speakers will decline even if the learning situation improves.

This study distinguished motivational intensity from motivation and explored its importance in EFL learning, which enriched the theoretical study of motivation theory and provided new evidence for EFL study. In addition, individual differences in the factors affecting EFL learners’ learning efforts were analyzed, which is conducive to clarifying the role of multiple factors in EFL learning and providing teaching suggestions for improving EFL learning. An appropriate amount of motivational intensity is conducive to developing the learning situation, increasing students’ interest, and maintaining the learning situation to improve personal attitudes toward English learning. The internal and external factors that affect the motivational intensity can keep it at a high level for a long time, thus improving students’ motivation and promoting English learning achievement. The SEM model between motivational intensity and learning motivation was established and analyzed, providing new ideas for studying EFL students.

However, some questions remain unanswered. For example, apart from personal, family and school factors, are there others that affect EFL students’ motivation? In addition, does motivational intensity show different states at different learning stages? These questions call for further study.

## Data availability statement

The original contributions presented in the study are included in the article/supplementary material, further inquiries can be directed to the corresponding author.

## Ethics statement

The studies involving human participants were reviewed and approved by the Human Research Ethics Program at the Education University of Hong Kong. The participants provided their written informed consent to participate in this study.

## Author contributions

CW: research design, data collection and analysis, and research implementation. SZ and HZ: data analysis. All authors contributed to the article and approved the submitted version.
